# The combined effects of physical frailty and cognitive impairment on emergency department- versus direct-admission hospitalizations

**DOI:** 10.1186/s12877-022-03397-6

**Published:** 2022-08-31

**Authors:** Brian Buta, Ari B. Friedman, Shang-En Chung, Orla C. Sheehan, Marcela D. Blinka, Susan L. Gearhart, Qian-Li Xue

**Affiliations:** 1 Department of Medicine Division of Geriatric Medicine and Gerontology, Center On Aging and Health, Johns Hopkins University, 2024 E. Monument Street, Suite 2-700, Baltimore, MD 21205 USA; 2 Department of Emergency Medicine, Perelman School of Medicine, University of Pennsylvania, PA Philadelphia, USA; 3 Connolly Hospital Blanchardstown, Dublin, Ireland

**Keywords:** Frailty, Cognition, Health services, Hospitalizations

## Abstract

**Background:**

We aimed to study whether physical frailty and cognitive impairment (CI) increase the risk of recurrent hospitalizations in older adults, independent of comorbidity, and disability.

**Methods:**

Two thousand five hundred forty-nine community-dwelling participants from the National Health and Aging Trends Study (NHATS) with 3 + years of continuous Medicare coverage from linked claims data were included. We used the marginal means/rates recurrent events model to investigate the association of baseline CI (mild CI or dementia) and physical frailty, separately and synergistically, with the number of all-source vs. Emergency Department (ED)-admission vs. direct admission hospitalizations over 2 years.

**Results:**

17.8% of participants had at least one ED-admission hospitalization; 12.7% had at least one direct admission hospitalization. Frailty and CI, modeled separately, were both significantly associated with risk of recurrent all-source (Rate Ratio (RR) = 1.24 for frailty, 1.21 for CI; *p* < .05) and ED-admission (RR = 1.49 for frailty, 1.41 for CI; *p* < .05) hospitalizations but not direct admission, adjusting for socio-demographics, obesity, comorbidity and disability. When CI and frailty were examined together, 64.3% had neither (Unimpaired); 28.1% CI only; 3.5% Frailty only; 4.1% CI + Frailty. Compared to those Unimpaired, CI alone and CI + Frailty were predictive of all-source (RR = 1.20, 1.48, *p* < .05) and ED-admission (RR = 1.36, 2.14, *p* < .05) hospitalizations, but not direct admission, in our adjusted model.

**Conclusions:**

Older adults with both CI and frailty experienced the highest risk for recurrent ED-admission hospitalizations. Timely recognition of older adults with CI and frailty is needed, paying special attention to managing cognitive impairment to mitigate preventable causes of ED admissions and potentiate alternatives to hospitalization.

**Supplementary Information:**

The online version contains supplementary material available at 10.1186/s12877-022-03397-6.

## Introduction

The medical and economic burden of caring for older adults with physical and cognitive deficits is a major challenge to healthcare systems. Accurate prediction of healthcare utilization is important for managed care organizations such as the U.S. federal health insurance program, Medicare, which serve a disproportionally high percentage of vulnerable older adults [1]. For older patients, hospitalizations represent important health events that can lead to sarcopenia and delirium, with long-term consequences for future functional impairments, as well as significant health system and out-of-pocket costs [2–5]. Hospitalizations resulting from Emergency Department (ED) evaluation are more typically due to an acute health stressor such as an infection, infarction, or fall than are hospitalizations in which the patient is directly admitted for a planned surgery, procedure, or monitoring [6].

Frailty and cognitive impairment are common health problems among older adults that can predict poor health outcomes including hospitalizations and death [7–10]. These conditions often co-exist: in a recent study, 67% of a US sample with frailty had cognitive impairment, while 28% of those with cognitive impairment also had frailty [7]. In previous studies, frailty and cognitive impairment/dementia have independently been associated with greater healthcare costs and hospitalizations [11–15]. However, there is limited research on the combined effects of cognitive impairment and physical frailty on hospitalizations. Therefore, we aimed to investigate the association of baseline cognitive impairment and physical frailty, separately and synergistically, with the number of all-source hospitalizations over 2 years in a large US nationally-representative sample linked to systematic health care data. We hypothesized that the presence of physical frailty and cognitive impairment, alone or combined, would increase the risk of recurrent hospitalizations in older adults, independent of socio-demographic factors, comorbidity, and disability. Additionally, we assessed the degree to which the relationships depended on the source of admission, i.e., ED-admission vs. direct admission hospitalizations.

## Methods

Our study sample draws from the National Health and Aging Trends Study (NHATS, www.nhats.org) [16], a nationally representative study of US Medicare beneficiaries ages 65 years and older, with NHATS data linked to Medicare claims [17]. The sampling probabilities of the original NHATS cohort were designed to yield equal probability samples by age group and race/ethnicity. This linked data set was available for approved use (Johns Hopkins Medicine IRB00077995). Our analytic sample included 2,549 community-dwelling older adults from the first wave of NHATS in 2011 (i.e., baseline visit), who had frailty and cognition measured at baseline, and were continuously enrolled in fee-for-service Medicare for at least 1 year prior and 2 years after baseline visit. Given that Medicare enrollees could enter and exit the program at any time for different reasons, the two-year study follow-up was chosen to maximize sample size and data capture of hospitalization events while also considering the availability of the Medicare claims data at the time of the analysis. Supplemental Fig. 1 displays exclusions: non-continuous Medicare coverage over this 3-year period; any hospitalization 1 year prior to baseline, which may exacerbate risk for further hospitalizations; and history of stroke or depression, due to possible overlapping symptoms with frailty or cognitive impairment [18].

Study variables included the physical frailty phenotype, as measured in NHATS, with 5 main criteria: exhaustion, low physical activity, slowness, weakness, and weight loss [19]. In brief, participants met criteria as follows: exhaustion, by self-reporting low energy or ease of exhaustion for limiting activities; low activity, by self-reporting never walking for exercise or engaging in vigorous activities; slowness, by walking speed (first of 2 tests of 3 m walk) at or below the 20^th^ percentile of the population distribution by sex and height categories; weakness, by grip strength (maximal value of two tests using a handheld dynamometer) at or below the 20^th^ percentile of the population distribution by sex and body mass index (BMI) categories; and weight loss, by BMI < 18.5 kg/m^2^, or reported unintentional loss of ≥ 10 pounds in the last year. Those meeting 3 + criteria were classified as frail; 0–2 as not-frail.

Cognitive impairment was determined by meeting at least one of three criteria [7]: 1) scored in bottom quintile in either executive function (clock-drawing test) or memory (10-item immediate and delayed recall batteries); 2) self- or proxy report of doctor’s diagnosis of dementia or Alzheimer’s disease (AD); or 3) scored of 2 or higher on the AD8 Dementia Screening Interview [20]. Probable dementia was defined as having met at least one of three criteria: [21] (1) self-respondents’ test performance less than 1.5 SD below the mean in at least two of three domains: memory, orientation, and executive functioning; (2) self- or proxy-report of dementia or AD by doctor’s diagnosis; (3) a score of 2 or higher on the AD8 administered to proxy respondents [22]. The first item in the probable dementia definition is distinct from our cognitive impairment definition; the other two items are the same. CI intends to capture a broader spectrum of cognitive impairment that includes probable dementia.

Covariates included age (years, in 5-year increments); race/ethnicity: (white non-Hispanic, Black non-Hispanic, Hispanic, or other); education: (eighth grade or less, ninth to twelfth grade (no diploma), or high school graduate or higher); and total personal annual income (income quartiles, < $15,000, $15,000–$30,000, $30,000–$60,000, or > $60,000 USD per year).

Health characteristics included body mass index (BMI), categorized as underweight (< 18.5 kg/m2), normal (≥ 18.5 and < 25 kg/m2), overweight (≥ 25 and < 30 kg/m2), or obese (≥ 30 kg/m2). Comorbidities included history of heart disease, hypertension, arthritis, osteoporosis, diabetes mellitus type II, lung disease, cancer, and hip fracture; and comorbidity burden by the number of chronic diseases (0,1,2,3,4 +). Activities of daily living (ADLs) (using the toilet, getting cleaned up, dressing, and eating) and Mobility Disability (getting out of bed, going outside, and getting around inside) were scored using an ordinal scale (“fully able,” “modification,” “difficulty,” or “assistance”), with dependency defined as requiring “assistance” [23, 24].

Outcomes included all-source hospitalizations; ED-admission hospitalizations, where an ED visit directly preceded hospitalization (typically unplanned, acute hospitalizations); and direct admission hospitalizations, where hospitalization occurred without a preceding ED visit (typically planned procedures). ED-admission hospitalizations were defined as inpatient claims with revenue codes 0450, 0451, 0452, 0456, 0459 and 0981. Centers for Medicare & Medicaid Services (CMS) data identifying hospitalizations after ED visits typically excludes ED visits within 24 h of another ED visit, to avoid double-counting via clerical errors, and considers a hospitalization to have resulted from an ED visit if it occurred on the same day or the next day compared to the ED visit, to allow for ED visits that crossed midnight before the admissions decision was made. Inpatient claims without revenue codes 0450, 0451, 0452, 0456, 0459 and 0981 were defined as direct admission hospitalizations. We defined a recurrent event as one or more hospitalizations of the same study subject within the two years following baseline assessment.

Statistical analyses: Descriptive statistics on sociodemographic and health characteristics were reported by analytic groups: 1) Unimpaired: those with no frailty or cognitive impairment; 2) Cognitive Impairment (CI) only: those with CI but not frailty; 3) Frailty only: those who were frail but not cognitively impaired; 4) CI + Frailty: participants who were both cognitively impaired and frail. In addition, we also compared the baseline characteristics of study subjects who had at least one hospitalization during the two-year follow-up by type of their first hospital admission (i.e., direct-admission vs. Emergency Department (ED)-admission). Frequency percentages were used to summarize categorical variables. Chi-square and Kruskal–Wallis tests were used to assess the difference in sociodemographic and health factors among CI/frailty groups. Our analyses focused on the association of baseline CI and physical frailty, separately and jointly, with the recurrence of all-source vs. ED-admission vs. directly admitted hospitalizations over 2 years analyzed in separate models using linked NHATS and Medicare claims data. We used a recurrent events model, the marginal means/rates model, where all-source, ED and direct admission hospitalization were treated as recurrence event outcomes with effect size reported as a rate ratio (RR). This approach considers all hospitalizations of the same subject as a single counting process and corrects for dependency among recurrent event times within a subject without the need to parameterize the dependence structure, therefore making it particularly appealing to applications where the dependence structure is complex and unknown, or the nature of the dependence is not of primary interest [25]. We first performed an unadjusted recurrent events model to examine the association between CI/Frailty groups and the risk of recurrent hospitalizations (analytic model 1). Secondly, we performed an adjusted model adding covariates: age (continuous), gender, race/ethnicity, education, income, and obesity (analytic model 2). Lastly, additional covariates of comorbidities (continuous) and dependency were included to assess the associations independent of multimorbidity and disability (analytic model 3). To examine the reasons for direct-admission vs. ED-admission hospitalizations, we analyzed the primary/principal diagnosis code established to be chiefly responsible for ED- or direct-admission hospitalizations. Diagnoses were classified by body system or condition using chapters from the International Classification of Diseases, ninth Revision, Clinical Modification (http://www.icd9data.com/2015/Volume1/default.htm). Percentage distribution of primary diagnosis code categories with the highest proportions (≥ 20%) of use among individuals who had one or more hospitalizations was tabulated by hospital admission type and by Frailty/CI group membership. Statistical analyses were performed using SAS (v.9.4; SAS Institute Inc, Cary, North Carolina) and Stata (v.15; StatCorp LLC, College Station, Texas). A *p*-value < 0.05 was considered statistically significant.

## Results

In our analytic sample of 2,549 participants, 1,640 (64.3%) were Unimpaired, 715 (28.1%) had CI only, 90 (3.5%) had Frailty only, and 104 (4.1%) had CI + Frailty. The sample was 75.6% white, 17.6% Black, and 6.8% Hispanic or another race/ethnicity; 57% were female. See Table 1: we found statistically significant differences across groups for age, sex, race/ethnicity, education, income, BMI, comorbidity, and disability (*p* = < 0.05). In general, our combined CI + Frailty group was older and had the greatest majority of non-white participants when compared the other groups. Participants with CI + Frailty also generally had lower income levels and less education than the other groups (46.2% of those with CI + Frailty were in the lowest income bracket compared to 17.9–34.4% in other groups; and 31.1% of CI + Frailty group had 8^th^ grade education or less compared to 4.8–16.6% among other groups), and had higher BMI levels that were comparable to Frailty only group (33.7% of Frailty only and 29.6% of CI + Frailty group were obese). More than half of those with CI + Frailty had probable dementia, compared to about 19% in the CI only group. Participants with CI + Frailty had worse health status compared to other groups, with a high percent of comorbidities similar to the Frailty only group (31.1% and 26.0% with 4 + comorbidities among Frailty only and CI + Frailty groups, respectively, compared to 12.3–14.1% among other groups). Both types of dependency – ADL and mobility disability – were highest in the combined group.

**Table 1:** Characteristics of study analytic sample at study baseline (Year 2011)

Variable	Overall *n* = 2549	Unimpaired *n* = 1640	Cognitive impairment only *n* = 715	Frailty only *n* = 90	Cognitive impairment + Frailty *n* = 104	*p*-value*
Age, mean (s.d.)	77.2 (7.4)	75.5 (6.7)	80.0 (7.8)	79.3 (7.8)	83.1 (7.2)	< .001
Sex, n (%)						0.057
Female	1449 (56.9)	920 (56.1)	401 (56.1)	61 (67.8)	67 (64.4)	
Male	1100 (43.2)	720 (43.9)	314 (43.9)	29 (32.2)	37 (35.6)	
Race/Ethnicity, n (%)						< .001
White non-Hispanic	1927 (75.6)	1335 (81.4)	475 (66.4)	62 (68.9)	55 (52.9)	
Black non-Hispanic	448 (17.6)	227 (13.8)	165 (23.1)	20 (22.2)	36 (34.6)	
Hispanic	100 (3.9)	41 (2.5)	46 (6.4)	**	**	
Other	74 (2.9)	37 (2.3)	29 (4.1)	**	**	
Education, n (%)						< .001
8^th^ grade or less	237 (9.3)	79 (4.8)	118 (16.6)	8 (8.9)	32 (31.1)	
9^th^-12^th^ grade (no diploma)	285 (11.2)	153 (9.4)	94 (13.2)	14 (15.6)	24 (23.3)	
High school graduate or higher	2019 (79.5)	1404 (85.8)	500 (70.2)	68 (75.6)	47 (45.6)	
Income, n (%)						< .001
Less than $15,000	604 (23.7)	294 (17.9)	231 (32.3)	31 (34.4)	48 (46.2)	
$15,000—$30,000	643 (25.3)	370 (22.6)	214 (29.9)	30 (33.3)	29 (27.9)	
$30,000 – $60,000	700 (27.5)	507 (30.9)	160 (22.4)	16 (17.8)	17 (16.4)	
More than $60,000	602 (23.6)	469 (28.6)	110 (15.4)	13 (14.4)	**	
BMI, n (%)						< .001
Underweight	41 (1.7)	14 (0.9)	15 (2.2)	**	**	
Normal	859 (34.8)	512 (32.1)	285 (41.4)	27 (30.3)	35 (35.7)	
Overweight	926 (37.5)	642 (40.2)	230 (33.4)	27 (30.3)	27 (27.6)	
Obese	645 (26.1)	428 (26.8)	158 (23.0)	30 (33.7)	29 (29.6)	
Probable dementia, n (%)	197 (7.7)	0	139 (19.4)	0	58 (55.8)	< .001
History of heart disease, n (%)	382 (15.0)	242 (14.8)	89 (12.5)	27 (30.0)	24 (23.1)	< .001
History of hypertension, n (%)	1613 (63.3)	1026 (62.6)	449 (62.8)	63 (70.0)	75 (72.1)	0.122
History of arthritis, n (%)	1322 (51.9)	837 (51.1)	345 (48.3)	65 (72.2)	75 (72.1)	< .001
History of osteoporosis, n (%)	492 (19.4)	310 (19.0)	133 (18.7)	24 (26.7)	25 (24.0)	0.204
History of diabetes, n (%)	513 (20.1)	303 (18.5)	155 (21.7)	23 (25.6)	32 (30.8)	0.007
History of lung disease, n (%)	287 (11.3)	178 (10.9)	66 (9.2)	23 (25.6)	20 (19.2)	< .001
History of cancer, n (%)	681 (26.7)	468 (28.5)	164 (22.9)	25 (27.8)	24 (23.1)	0.031
History of hip fracture, n (%)	84 (3.3)	35 (2.1)	38 (5.3)	**	**	< .001
Number of diseases, n (%)						< .001
0	278 (10.9)	194 (11.8)	80 (11.2)	**	**	
1	580 (22.8)	363 (22.1)	193 (27.0)	12 (13.3)	12 (11.5)	
2	742 (29.1)	496 (30.2)	190 (26.6)	21 (23.3)	35 (33.7)	
3	575 (22.6)	356 (21.7)	164 (22.9)	26 (28.9)	29 (27.9)	
4 +	374 (14.7)	231 (14.1)	88 (12.3)	28 (31.1)	27 (26.0)	
Activities of daily living (ADLs), n (%)						< .001
Fully able for all activities	1113 (43.7)	807 (49.2)	269 (37.6)	17 (18.9)	20 (19.2)	
Modification in any activity	1162 (45.6)	738 (45.0)	342 (47.8)	44 (48.9)	38 (36.5)	
Difficulty in any activity	94 (3.7)	47 (2.9)	29 (4.1)	**	**	
Assistance in any activity	180 (7.1)	48 (2.9)	75 (10.5)	19 (21.1)	38 (36.5)	
Mobility disability, n (%)						< .001
Fully able for all activities	1860 (73.0)	1352 (82.4)	468 (65.5)	20 (22.2)	20 (19.2)	
Modification in any activity	376 (14.8)	199 (12.1)	128 (17.9)	27 (30.0)	22 (21.2)	
Difficulty in any activity	109 (4.3)	45 (2.7)	35 (4.9)	15 (16.7)	14 (13.5)	
Assistance in any activity	204 (8.0)	44 (2.7)	84 (11.8)	28 (31.1)	48 (46.2)	

Note. ** represent cell sizes of 11 or less, per National Institute on Aging CMS data cell size suppression

^*^p-value is determined by Chi-Square for categorical variables or Kruskal–Wallis tests for continuous variables

**Fig. 1:**
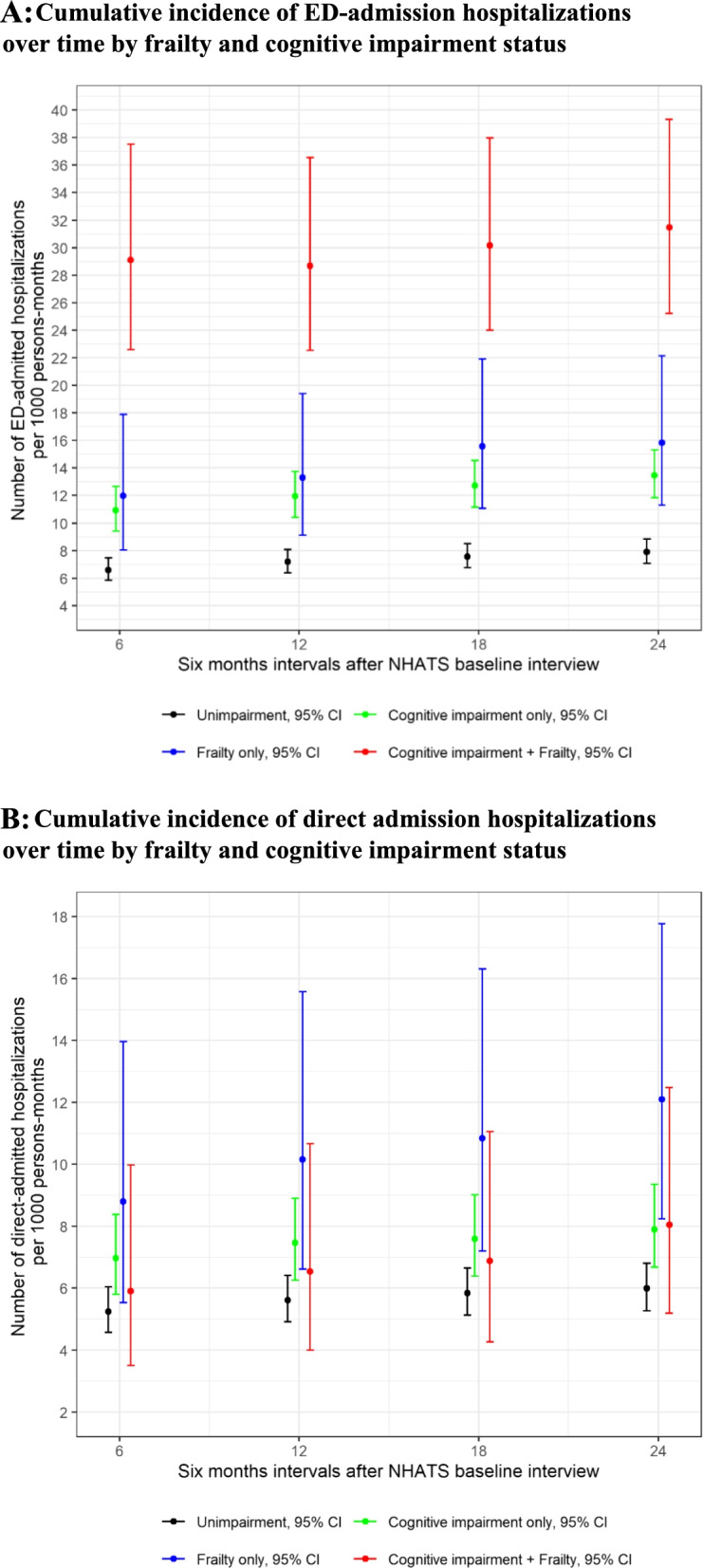
**A** Cumulative incidence of ED-admission hospitalizations over time by frailty and cognitive impairment status. **B** Cumulative incidence of direct admission hospitalizations over time by frailty and cognitive impairment status

During the 2-year period following baseline, 651 subjects had at least one hospitalization; and the total number of ED-admission hospitalizations was 654 and the total number of direct admission hospitalizations was 416. 17.8% (*n* = 455) of our 2,549 study participants had at least one ED-admission hospitalization, and 12.7% (*n* = 323) had at least one direct hospitalization. Those whose 1^st^ episode of hospitalization was ED-admission hospitalization were more like to have probable dementia (17.7% vs. 8.3%, *p* < 0.001) and mobility dependency (15.1% vs. 8.7%, *p* = 0.020), but less likely to be overweight or obese (58.3% vs. 70.2%, *p* = 0.024) and less likely to have arthritis (57.6% vs. 66.4%, *p* = 0.026) than those whose 1^st^ episode of hospitalization was direct-admission hospitalization (Supplemental Table). Figures 1A and 1B show cumulative incidence of ED-admission and direct admission hospitalizations over time by frailty and cognitive impairment status. While those with cognitive impairment and frailty had the highest cumulative incidence of ED-admission hospitalizations; those with frailty alone had the highest cumulative incidence of direct admission hospitalizations.

Table 2 presents the results of our recurrent events analyses for all-cause hospitalizations. In our Frailty model, when compared to not-frail participants, frail participants had a significantly increased risk for all-source hospitalizations (Rate Ratio (RR) = 1.42, 95% Confidence Interval (95%CI) = 1.19,1.70) in analytic model 2, adjusted for socio-demographics and BMI. In our CI model, those with CI versus no CI also had significantly increased risk for all-source hospitalizations (RR = 1.20, 95%CI = 1.04,1.39) in analytic model 2. In our Combined model, with Unimpaired participants as the reference group, CI only (RR = 1.19, 95%CI = 1.05,1.42), Frailty only (RR = 1.39, 95%CI = 1.05,1.85), and the combined CI + Frailty (RR = 1.68, 95%CI = 1.32,2.13) groups were significantly associated with all-source hospitalizations in this model. However, in analytic model 3, controlled for comorbidity and disability, the Frailty only group no longer had a statistically significant association.

**Table 2:** Rate ratios (95% confidence intervals) for all-cause hospitalizations during 2-year follow-up period, by frailty/cognition status at baseline

	**Analytic Model 1**^**a**^	**Analytic Model 2**^**b**^	**Analytic Model 3**^**c**^
**Frailty Model**			
Not Frail (Reference)	1.00	1.00	1.00
Frail	1.70 (1.43,2.01)	1.42 (1.19,1.70)	1.24 (1.03,1.50)
**Cognitive Impairment Model**			
No Cognitive Impairment (Reference)	1.00	1.00	1.00
Cognitively impaired (CI)	1.43 (1.26,1.63)	1.20 (1.04,1.39)	1.21 (1.04,1.39)
**Combined Model**			
Unimpaired (Reference)	1.00	1.00	1.00
CI only	1.38 (1.20,1.59)	1.19 (1.02,1.38)	1.20 (1.03,1.40)
Frailty only	1.61 (1.21,2.13)	1.39 (1.05,1.85)	1.23 (0.92,1.89)
CI + Frailty	2.14 (1.73,2.64)	1.68 (1.32,2.13)	1.48 (1.16,1.89)

^a^ unadjusted

^b^ controlled for age (continuous), gender, race/ethnicity, education, income, obesity

^c^ controlled for model 2 + comorbidity (continuous), dependency

Table 3 presents the results for ED-admission hospitalizations. In the Frailty model, frail participants had significantly increased risk for ED-admission hospitalizations in analytic model 2; and in the CI model, those with CI also had significantly increased risk. In the Combined model, CI only (RR = 1.35, 95%CI = 1.07,1.69) and the combined CI + Frailty group were (RR = 2.55, 95%CI = 1.77,3.68) significantly associated with ED-admission hospitalizations. The Frailty only group was not. This pattern was sustained in analytic model 3, adjusting for comorbidity and disability.

**Table 3:** Rate ratios (95% confidence intervals) for emergency department (ED)-admission hospitalizations during 2-year follow-up period, by frailty/cognition status at baseline

	**Analytic Model 1**^**a**^	**Analytic Model 2**^**b**^	**Analytic Model 3**^**c**^
**Frailty Model**			
Not Frail (Reference)	1.00	1.00	1.00
Frail	2.53 (1.95,3.28)	1.80 (1.37,2.38)	1.49 (1.13,1.97)
**Cognitive Impairment Model**			
No Cognitive Impairment (Reference)	1.00	1.00	1.00
Cognitively impaired (CI)	1.90 (1.56,2.30)	1.42 (1.14,1.76)	1.41 (1.14,1.74)
**Combined**			
Unimpaired (Reference)	1.00	1.00	1.00
CI only	1.70 (1.38,2.11)	1.35 (1.07,1.69)	1.36 (1.08,1.70)
Frailty only	2.00 (1.29,3.10)	1.55 (0.99,2.41)	1.30 (0.82,2.08)
CI + Frailty	3.99 (2.91,5.47)	2.55 (1.77,3.68)	2.14 (1.50,3.06)

^a^ unadjusted

^b^ controlled for age (continuous), gender, race/ethnicity, education, income, obesity

^c^ controlled for model 2 + comorbidity (continuous), dependency

Table 4 presents the results of our analyses for direct-admission hospitalizations. In both the Frailty and CI models, we did not see significant associations between these conditions and directly admitted hospitalizations in analytic model 2. In our Combined model, the Frailty only group had a statistically significant association with recurrent direct-admission hospitalization (RR = 1.98, 95%CI = 1.10,3.55) in analytic model 2; there was also a 69% increased risk for direct hospitalization in analytic model 3 for Frailty only, though not statistically significant.

**Table 4:** Rate ratios (95% confidence intervals) for direct-admission hospitalizations during 2-year follow-up period, by frailty/cognition status at baseline

	**Analytic Model 1**^**a**^	**Analytic Model 2**^**b**^	**Analytic Model 3**^**c**^
**Frailty Model**			
Not Frail (Reference)	1.00	1.00	1.00
Frail	1.51 (0.98,2.32)	1.42 (0.88,2.28)	1.22 (0.78,1.90)
**Cognitive Impairment Model**			
No Cognitive Impairment (Reference)	1.00	1.00	1.00
Cognitively impaired (CI)	1.26 (0.98,1.60)	1.17 (0.88,1.56)	1.18 (0.88,1.59)
**Combined**			
Unimpaired (Reference)	1.00	1.00	1.00
CI only	1.32 (1.02,1.70)	1.26 (0.94,1.69)	1.28 (0.95,1.72)
Frailty only	2.02 (1.13,3.61)	1.98 (1.10,3.55)	1.69 (0.97,2.96)
CI + Frailty	1.34 (0.72,2.51)	1.20 (0.58,2.52)	1.04 (0.50,2.17)

^a^ unadjusted

^b^ controlled for age (continuous), gender, race/ethnicity, education, income, obesity

^c^ controlled for model 2 + comorbidity (continuous), dependency

Table 5 shows that the three most common primary diagnoses classified by body system or condition for the direct-admission hospitalizations were Circulatory system, Musculoskeletal system, and Symptoms, Signs, and Ill-Defined Conditions; and the top three for the ED-admission hospitalizations were Symptoms, Signs, and Ill-Defined Conditions, Circulatory system, and Respiratory system. Within the direct-admission hospitalization type, Circulatory system was the leading primary diagnosis among the unimpaired, the CI only, and the combined CI and Frailty; whereas Symptoms, Signs, and Ill-Defined Conditions were the most common for the Frailty only group. Within the ED-admission hospitalization type, the top three primary diagnoses were the same for all CI/Frailty groups except the combined CI and Frailty where Genitourinary system became the 3rd most common primary diagnosis as in the case of the direct-admission hospitalizations. It is also worth noting that Musculoskeletal system and Injury were among the leading common primary diagnoses for the combined CI and Frailty group regardless of admission type.

**Table 5:** Percentage distribution^a^ of primary admission diagnosis code category by body system or condition^b^ with highest proportions (≥ 20%) of use among individuals who had one or more hospitalizations

Direct-admission Hospitalizations							
Unimpaired	Circulatory	Musculoskeletal	Symptoms				
(*n* = 187)	(41.2)	(36.4)	(27.8)				
Cognitive impairment (CI) only	Circulatory	Symptoms	Musculoskeletal				
(*n* = 105)	(46.7)	(38.1)	(32.4)				
Frailty only	Symptoms	Circulatory	Musculoskeletal	Respiratory			
(*n* = 18)	(44.4)	(38.9)	(28.0)	(27.8)			
CI + Frailty	Circulatory	Symptoms	Genitourinary	Injury	Musculoskeletal	Digestive	
(*n* = 13)	(61.5)	(38.5)	(30.8)	(23.1)	(23.1)	(23.1)	
**Emergency Department (ED)-admission Hospitalizations**							
Unimpaired	Symptoms	Circulatory	Respiratory	Digestive			
(*n* = 226)	(80.5)	(55.8)	(28.3)	(22.6)			
CI only	Symptoms	Circulatory	Respiratory	Injury	Musculoskeletal	Digestive	
(*n* = 158)	(80.4)	(54.4)	(29.8)	(25.9)	(23.4)	(22.2)	
Frailty only	Symptoms	Circulatory	Respiratory	Digestive			
(*n* = 22)	(86.4)	(81.8)	(27.3)	(27.3)			
CI + Frailty	Symptoms	Circulatory	Genitourinary	Musculoskeletal	Respiratory	Digestive	Injury
(*n* = 49)	(89.8)	(51.0)	(36.7)	(28.6)	(22.5)	(20.4)	(20.4)

^a^ the primary admission diagnosis code could have more than one, i.e., not mutually exclusive. As such, the row total of the percentage values tabulated is not expected to be 100%

^b^ using diagnostic codes from the International Classification of Diseases, ninth Revision, Clinical Modification (ICD-9-CM) (http://www.icd9data.com/2015/Volume1/default.htm): Diseases Of The Circulatory System (“Circulatory”; ICD-9-CM codes: 390–459), Symptoms, Signs, And Ill-Defined Conditions (“System”; ICD-9-CM codes: 780–799), Diseases Of The Musculoskeletal System And Connective Tissue (“Musculoskeletal”; ICD-9-CM codes: 710–739), Diseases Of The Respiratory System (“Respiratory”; ICD-9-CM codes: 460–519), Diseases Of The Genitourinary System (“Genitourinary”; ICD-9-CM codes: 580–629), Injury And Poisoning (“Injury”; ICD-9-CM codes: 800–999), Diseases Of The Digestive System (“Digestive”; ICD-9-CM codes: 520–579)

## Discussion

When compared to those with neither CI nor frailty, CI + Frailty was predictive of all-source and ED-admission hospitalizations, but not directly admitted hospitalizations, after adjusting for socio-demographics, BMI, comorbidity and disability. Older adults with both CI and physical frailty were the most at-risk group for ED-admission hospitalizations during the 2-year follow-up period. Those with CI only were also at higher risk of recurrent ED-initiated hospitalizations. Promoting strategies for ED avoidance could help to prevent ED-admission hospitalizations in these high-risk patients [14].

In our study, participants with Cognitive Impairment only, Frailty only, and CI + Frailty had a significantly higher risk of recurrent all-source hospitalizations, compared to our Unimpaired reference group. This finding highlights that frailty and CI are both independently associated with increased all-source hospitalizations, and the risk was the highest when both were present, though there was not a significant synergistic effect between frailty and CI. In analytic model 3, Frailty only was no longer statistically significant, likely due to over-adjustment as comorbidity and dependency may be precursors or antecedents of frailty in a causal relationship.

In previous studies, frailty and CI/dementia have been separately shown to have independent associations with greater healthcare utilization and hospitalizations. Ensrud and colleagues reported statistically significant increased odds of hospitalizations using claims data among frail women and frail older men [11, 12]. Phelan and colleagues reported higher all-cause hospital admission rates among older adults with dementia compared to those without dementia [15]. One study showed that patients with Alzheimer’s disease were more likely to experience hospitalizations and emergency department visits than matched controls several years before AD diagnosis [14].

Few studies have looked into both physical and cognitive measures and healthcare utilization. In the Study of Osteoporotic Fractures, older women who had both poor mobility and dementia had higher healthcare utilization compared to those with good mobility and normal cognition [13]. One recent study examined the effects of concomitant cognitive impairment (defined by Mini-Mental Status Exam (MMSE) score) and frailty (using the PFP) on self-reported admission(s) to the hospital. The study found an odds ratio of 6.57 for any hospitalization in the combined group compared to the group with neither frailty nor cognitive impairment [26]. In another recent study, community-dwelling Chinese adults in the US with both cognitive deficits (using MMSE) and physical frailty (defined by Short Physical Performance Battery (SPPB)) had increased likelihood of self-reported hospitalizations and emergency department visits [27]. In that context, contrary to our findings, those with isolated cognitive impairment did not have increased risks of hospitalization or ED visits, but those with physical frailty alone did. This difference may be due to the study’s population (sample of Chinese adults in the greater Chicago area), design (self-report healthcare utilization may be subject to recall bias) and measures (global cognitive function; SPPB for frailty). While frailty and CI are known risk factors for adverse health outcomes, our results add to the literature by highlighting the additive effect when frailty is combined with cognitive impairment, as well as by differentiating between ED-admission and direct-admission hospitalization, which may reflect entirely different phenomena given the central role of the modern ED in unscheduled admissions. The presence of frailty in particular may reduce the propensity of surgeons to operate (and therefore to admit directly to their hospital service) for a given surgical indication [28, 29]. Another possible scenario is that a surgeon operates on a directly-admitted frail patient, which may be followed by ED admission should the patient develop complications. These considerations appeal to the need for preventive strategies to avoid ED admission in vulnerable patients.

Frailty, CI, and combined CI + Frailty lead to increased ED-admission hospitalizations through a variety of mechanisms. First, these impairments likely increase the probability of an acute health shock. For instance, disabilities associated with frailty and impaired cognition such as gait disturbances may increase the probability of a fall [30, 31]. The observed higher rates of injury regardless of hospital admission type in the combined frailty and CI group provide empirical evidence for a greater incidence of trauma including injurious falls when both cognitive impairment and physical frailty are present. Similarly, cognitive impairment may cause difficulty managing medications and preventative care for chronic disease to prevent acute exacerbations [14], with greater challenges as the number of chronic diseases increase as in multimorbidity, which is more common in frail older adults [32]. Our study found that diseases of the respiratory system were among the leading primary diagnoses in the ED-admission group; and 26% of the respiratory diagnoses belong to the subcategory of pneumonia and influenza. Given that influenza is a common cause of pneumonia, active promotion of preventive measures such as influenza vaccination among older adults and vulnerable subsets in particular may prove to be effective at reducing incidence of potential preventable ED-admission hospitalization. The fact that the prevalence of probable dementia in the ED-admission group was twice as high as in the direct-admission group presents a unique challenge to the delivery of preventive care to those with cognitive impairment and the importance of aligning care with goals of patients and their families [33]. Second, frailty and CI separately and in combination may worsen the severity of acute health shocks. Frailty in particular may increase susceptibility to an acute health shock once it has occurred [34]. Cognitive impairment may impede a patient’s ability to report symptoms, leading to delayed presentation and therefore more severe illness, for instance delayed presentation for diverticulitis leading to an ED visit requiring hospitalization for perforation rather than discharge on oral antibiotics, or delayed presentation of pneumonia with delirium compounding dementia and worsening confusion in the ED. Third, without modifying the probability or severity of underlying disease, communication challenges in particular may increase the propensity to present to the ED or the probability of admission conditional on presenting to the ED. For instance, increased involvement of caregivers among frail and/or CI patients could mean more opportunities for recognition of hospitalization-requiring disease [35], and communication challenges in CI could lead to more opportunities for admission for vague diagnoses (e.g. “failure to thrive”) [36]. Communication challenges could also delay access to emergency care for those without involved caregivers.

Strengths of this study include a large nationally representative sample of older adults in the United States. We also used linked Medicare claims data, which provides an objective source of healthcare utilization information for older adults in the United States, although we recognize that Medicare claims data was not inherently designed for research purposes [37]. In addition, our assessment of cognitive impairment was based on self or proxy-report and cognition performance. Although we could have used claims-based dementia diagnosis, it would limit our ability to capture a broader spectrum of cognitive impairment as one past study showed that only half of patients clinically diagnosed with mild cognitive impairment had dementia diagnoses in Medicare claims [38]. Another recent study reported that those unaware of dementia diagnoses has similar risk for acute care utilization as those who were aware [39]. In this study, we modeled hospitalization incidence as a recurrent event using the marginal means/rates model that effectively accounted for repeated hospitalizations experienced by a study subject over time. As such, the analysis captures the impact of frailty and CI on not only any occurrence but also the frequency of hospitalization. It is also important to note that a unique contribution of our study is to distinguish ED-admission vs. direct-admission hospitalizations and the differential findings on the impact of physical frailty and cognitive impairment depending on the source of admission. Our study was also limited by the modest number of participants in our categories of Frailty only and CI + Frailty. This relative lack of power may explain why, despite a greater point estimate of relative risk of the frailty only group to that of the CI only group, the result was not significant in the model of ED-admission hospitalizations. However, even after adjustment and appropriately accounting for serial autocorrelation in the recurrent events, our analysis was able to demonstrate several findings that advance our understanding of how health risk and healthcare utilization are modified by frailty, CI, and their overlap, as well as the importance of considering the source of hospital admission in such studies.

## Conclusions

Our study found that not only frailty and CI were independently associated with all-cause hospitalizations, but also older adults with both CI and frailty experienced the highest risk for recurrent ED-admission hospitalizations, but not direct-admission hospitalizations. The clinical implications of these findings are twofold. First, screening for cognition and frailty at the time of hospital admission can be utilized as flags to prioritize care plans for these vulnerable patients. However, the implementation of the routine collection of measures of frailty and cognition in clinical settings, especially in hospitals and emergency departments, is an ongoing challenge due to a combination of factors including time and resource constraints and feasibility of objective measurement (e.g., gait speed). Future efforts should focus on validated methods that are quick, require minimal resources to collect, and/or draw from routinely collected health data. Second, building on this study, future studies with a large sample size should further explore the reasons for hospitalization, by ED or direct admission, and the relationship between these causes and physical frailty and/or CI. In addition, we recommend further study of preventable causes of hospitalizations. This may be especially true in the ED setting as it is becoming the primary source of hospitalization for older adults [40]. Ultimately, interventions designed to reduce the risk of hospitalization and iatrogenic harm resulting from hospitalization need to consider factors operating at the patient, provider, and health system levels and in different clinical settings.

## Supplementary Information

**Additional file 1.**

**Additional file 2.**

## Data Availability

The data that support the findings of this study are available from the National Health and Aging Trends Study (NHATS) but restrictions apply to the availability of these data, which were used under Data Use Agreements from NHATS and CMS for the current study, and so are not publicly available. NHATS- CMS linked Restricted Data files are available to qualified researchers by applying directly to NHATS and CMS. Interested researchers can please visit the Data Access section of the NHATS website for application information: https://www.nhats.org/researcher/data-access.

## Abbreviations

AD: Alzheimer’s disease
BMI: Body Mass Index
CI: Cognitive Impairment
CMS: Centers for Medicare & Medicaid Services
ED: Emergency Department
MMSE: Mini-Mental Status Exam
NHATS: National Health and Aging Trends Study
RR: Rate Ratio
SPPB: Short Physical Performance Battery
