# Supplementary material for: The combined effects of physical frailty and cognitive impairment on emergency department- versus direct-admission hospitalizations
Source: BMC Geriatr. 2022 Aug 31;22:718. doi: 10.1186/s12877-022-03397-6 (PMC9429704; doi:10.1186/s12877-022-03397-6)
Supplement: Supplementary file 1 — Additional file 1. [file 12877_2022_3397_MOESM1_ESM.docx]

**Supplemental Figure 1: Flow of inclusions and exclusions for the study analytic sample**

NHATS Baseline community dwelling or non-nursing home residents

n=7,497

Continuous Medicare coverage for 1 year prior to baseline and 2 years after baseline

n=3,876

No hospitalization one year prior to baseline visit

n=3,219

No history of stoke or depression

n=2,549
