# Supplementary material for: The combined effects of physical frailty and cognitive impairment on emergency department- versus direct-admission hospitalizations
Source: BMC Geriatr. 2022 Aug 31;22:718. doi: 10.1186/s12877-022-03397-6 (PMC9429704; doi:10.1186/s12877-022-03397-6)
Supplement: Supplementary file 2 — Additional file 2. [file 12877_2022_3397_MOESM2_ESM.docx]

**Supplemental Table** Baseline characteristics of study subjects who had at least one hospitalization during the two-year follow-up by type of their first hospital admission

| **Variable** | **Overall**  **n=651** | **Direct admission**  **n=241** | **ED admission**  **n=410** | **p-value^1^** |
| --- | --- | --- | --- | --- |
| Age, mean (s.d.) | 77.2 (7.4) | 79.3 (7.8) | 83.1 (7.2) | <.001 |
| Sex, n (%)  Female  Male | 364 (55.9)  287 (44.1) | 129 (53.5)  112 (46.5) | 235 (57.3)  175 (42.7) | 0.347 |
| Race/Ethnicity, n (%)  White non-Hispanic  Black non-Hispanic  Hispanic  Other | 499 (76.7)  110 (16.9)  21 (3.2)  21 (3.2) | 193 (80.1)  37 (15.4)  **  ** | 306 (74.6)  73 (17.8)  15 (3.7)  16 (3.9) | 0.323 |
| Education, n (%)  8^th^ grade or less  9^th^-12^th^ grade (no diploma)  High school graduate or higher | 68 (10.5)  82 (12.7)  496 (76.8) | 18 (7.5)  30 (12.5)  193 (80.1) | 50 (12.4)  52 (12.8)  303 (74.8) | 0.126 |
| Income, n (%)  Less than $15,000  $15,000 - $30,000  $30,000 – $60,000  More than $60,000 | 173 (26.6)  176 (27.0)  184 (28.3)  118 (18.1) | 54 (22.4)  71 (29.5)  71 (29.5)  45 (18.7) | 119 (29.0)  105 (25.6)  113 (27.6)  73 (17.8) | 0.303 |
| BMI, n (%)  Underweight  Normal  Overweight  Obese | 14 (2.2)  222 (35.2)  233 (36.9)  162 (25.7) | **  66 (28.6)  96 (41.6)  66 (28.6) | **  156 (39.0)  137 (34.3)  96 (24.0) | 0.024 |
| Probable dementia, n (%) | 92 (14.1) | 20 (8.3) | 72 (17.7) | <.001 |
| History of heart disease, n (%) | 146 (22.4) | 47 (19.5) | 99 (24.2) | 0.167 |
| History of hypertension, n (%) | 454 (69.9) | 158 (65.8) | 296 (72.2) | 0.090 |
| History of arthritis, n (%) | 395 (60.9) | 160 (66.4) | 235 (57.6) | 0.026 |
| History of osteoporosis, n (%) | 139 (21.5) | 48 (19.9) | 91 (22.4) | 0.463 |
| History of diabetes, n (%) | 158 (24.3) | 59 (24.5) | 99 (24.2) | 0.923 |
| History of lung disease, n (%) | 89 (13.7) | 35 (14.6) | 54 (13.2) | 0.614 |
| History of cancer, n (%) | 197 (30.3) | 76 (31.5) | 121 (29.5) | 0.588 |
| History of hip fracture, n (%) | 30 (4.6) | ** | 21 (5.1) | 0.414 |
| Number of diseases, n (%)  0  1  2  3  4+ | 41 (6.3)  108 (16.6)  183 (28.1)  180 (27.7)  139 (21.4) | 12 (5.0)  42 (17.4)  71 (29.5)  66 (27.4)  50 (20.8) | 29 (7.1)  66 (16.1)  112 (27.3)  114 (27.8)  89 (21.7) | 0.813 |
| Activities of daily living (ADLs), n (%)  Fully able for all activities  Modification in any activity  Difficulty in any activity  Assistance in any activity | 231 (35.5)  321 (49.3)  27 (4.2)  72 (11.1) | 88 (36.5)  126 (52.3)  **  18 (7.5) | 143 (34.9)  195 (47.6)  18 (4.4)  54 (13.2) | 0.128 |
| Mobility disability, n (%)  Fully able for all activities  Modification in any activity  Difficulty in any activity  Assistance in any activity | 399 (61.3)  127 (19.5)  42 (6.5)  83 (12.8) | 165 (68.5)  41 (17.0)  14 (5.8)  21 (8.7) | 234 (57.1)  86 (21.0)  28 (6.8)  62 (15.1) | 0.020 |

^1^p-value is determined by Chi-Square and Kruskal-Wallis tests

Note: ** represent cell sizes of 11 or less, per National Institute on Aging CMS data cell size suppression policy
